# Upregulation of DARS2 by HBV promotes hepatocarcinogenesis through the miR-30e-5p/MAPK/NFAT5 pathway

**DOI:** 10.1186/s13046-017-0618-x

**Published:** 2017-10-19

**Authors:** Xian Qin, Changsheng Li, Tao Guo, Jing Chen, Hai-Tao Wang, Yi-Tao Wang, Yu-Sha Xiao, Jun Li, Pengpeng Liu, Zhi-Su Liu, Quan-Yan Liu

**Affiliations:** 1 Department of General Surgery, Research Center of Digestive Diseases, Zhongnan Hospital of Wuhan University, Donghu Road 169, Wuhan, 430071 People’s Republic of China; 2 Department of Endocrinology, Zhongnan Hospital of Wuhan University, Wuhan, 430071 People’s Republic of China

**Keywords:** Hepatocellular carcinoma, HBV, NFAT5, miR-30e-5p, DARS2

## Abstract

**Background:**

Infection with the hepatitis B virus (HBV) is closely associated with the development of hepatocellular carcinoma (HCC). The osmoregulatory transcription factor nuclear factor of activated T-cells 5 (NFAT5) has been shown to play an important role in the development of many types of human cancers. The role of NFAT5 in HBV-associated HCC has never previously been investigated.

**Methods:**

We compared expression profiles of NFAT5, DARS2 and miR-30e-5p in HCC samples, adjacent nontumor tissues and different hepatoma cell lines by quantitative real-time polymerase chain reaction and /or Western blot. Clinical data of HCC patients for up to 80 months were analyzed. The regulatory mechanisms upstream and convergent downstream pathways of NFAT5 in HBV-associated HCC were investigated by ChIP-seq, MSP, luciferase report assay and bioinformation anaylsis.

**Results:**

We first found that higher levels of NFAT5 expression predict a good prognosis, suggesting that NFAT5 is a potential tumor-suppressing gene, and verified that NFAT5 promotes hepatoma cell apoptosis and inhibits cell growth in vitro. Second, our results showed that HBV could suppress NFAT5 expression by inducing hypermethylation of the AP1-binding site in the NFAT5 promoter in hepatoma cells. In addition, HBV also inhibited NFAT5 through miR-30e-5p targeted MAP4K4, and miR-30e-5p in turn inhibited HBV replication. Finally, we demonstrated that NFAT5 suppressed DARS2 by directly binding to its promoter. DARS2 was identified as an HCC oncogene that promotes HCC cell cycle progression and inhibits HCC cell apoptosis.

**Conclusion:**

HBV suppresses NFAT5 through the miR-30e-5p/mitogen-activated protein kinase (MAPK) signaling pathway upstream of NFAT5 and inhibits the NFAT5 to enhance HCC tumorigenesis via the downstream target genes of DARS2.

**Electronic supplementary material:**

The online version of this article (10.1186/s13046-017-0618-x) contains supplementary material, which is available to authorized users.

## Background

The development of hepatocellular carcinoma (HCC) is closely associated with infection with the hepatitis B virus (HBV) [1]. However, the molecular mechanisms involved in HBV-mediated hepatocarcinogenesis remain unclear. The current study suggests that osmotic stress plays an important role in the development of inflammation and tumors [2]. The liver, kidneys, gastrointestinal tract and other tissues and organs are significantly differentially affected by exposure to an environment with moderate changes in osmotic pressure [3]. In rat livers with hypotension, the liver cells swell and become hypotonic, which activates mitogen-activated protein kinase (MAPK) pathways and inhibits proteolysis [4]. However, liver cells continue to shrink and become dehydrated while in a hyperosmolar state, resulting in the activation of CD95 and the eventual induction of apoptosis [5]. Further research into the relationship between osmotic stress and apoptosis in liver cells indicated that hepatocyte apoptosis can be inhibited by activating the integrin/Src/p38 MAPK signaling pathways in cells in a hypotonic state, whereas hepatocyte apoptosis can be promoted by activating NOX/ROS/CD95 while cells are in a hyperosmolar state [6, 7]. The liver cells of patients with chronic hepatitis B virus infections reside in a long-term hypertonic environment resulting from inflammatory infiltration, which leads to the accumulation of mutations and malignant representation in liver cells. These phenomena result from the rapid cellular regeneration induced by the environment of sustained necrosis. Therefore, osmotic stress in liver tissues may be associated with the occurrence and development of HBV-associated HCC.

Nuclear factor of activated T-cells 5 (NFAT5), also known as tonicity-responsive enhancer-binding protein (TonEBP), is a transcription factor that is crucial for cellular responses to hypertonic stress [8]. Unlike other members of the NFAT family (NFAT1-NFAT4), NFAT5 is not regulated by Ca^2+^ and, thus, is defined as the calcium-independent phosphatase calcineurin [9]. Under high osmotic pressure, NFAT5 promotes the transcription of osmotic pressure protection genes, such as aldose reductase (AR), taurine transport protein (TauT), Na^+^-dependent myo-inositol cotransporter (SMIT) and heat shock protein 70 (HSP70), thereby protecting cells from injury resulting from high osmotic pressure [10–12]. It has been suggested that NFAT5 plays important roles in different cell types and tissues, in an at least partly tonicity-independent manner, during embryonic development, cell differentiation, inflammatory processes, and cellular stress responses [13]. Recent studies have shown that NFAT5 is involved in the pathogenesis of multiple cancers, including non-small cell lung cancer, leiomyoma, breast cancer, melanoma and renal carcinoma [14–17]. However, the role of NFAT5 in HBV-associated HCC has never previously been investigated.

In this study, we investigated the mechanism whereby HBV affects NFAT5 by separating the upstream pathway and convergent downstream pathways of NFAT5 in hepatoma cells. Our results showed that HBV could suppressed NFAT5 expression by inducing hypermethylation of the AP1-binding site in the NFAT5 promoter and inhibiting miR-30e-5p/MAP4K4/DARS2 pathway in hepatoma cells. DARS2, as a downstream target gene of NFAT5, promoted HCC tumorigenesis by accelerating cell cycle progression and attenuating cell apoptosis. Our findings provide a novel biomarker for early HCC diagnosis and a novel therapeutic target in HCC.

## Methods

### Patient samples and clinical data

HCC tissues were collected in our hospital during hepatectomies of HCC patients who were first diagnosed with HCC and had not received any prior treatment. The patients and their families agreed to donate the HCC tissue for our research. The HCC tissues were collected and immediately stored at −80 °C in an RNA protective reagent. Clinical data were collected from EMR(electronic medical record) of our department. Detailed clinical data are listed in Table 1 and Additional file 1: Table S1. Follow-up visits for the survival analysis occurred for up to 80 months for NFAT5, or 40 months for DARS2 until recurrence or death.

### Cell culture and transfection

Cell lines, including the human normal liver cell L02 and the hepatoma cell lines Huh 7, HepG2 and Hep3B cells were obtained from the Cell Bank of the Shanghai Institute of Cell Biology at the Chinese Academy of Science (Shanghai, China), where they were characterized by mycoplasma detection, DNA fingerprinting, isozyme detection, and determination of cell viability. The HepG2.2.15 cell line was derived from HepG2 cells and stably expresses HBV(Genotype D, Serotype*ayw*, U95551), which was used as an HBV replication model. The stable cell lines were maintained in DMEM containing 400 μg/ml G418. The plasmid pCMV-HBV-1.3, which expresses HBV (genotype C, serotype *adr*, FJ899793), was a gift from Dr. Ying Zhu (State Key Laboratory of Virology, College of Life Sciences, Wuhan University, China). All cells were incubated at 37 °C in a humidified atmosphere containing 5% CO_2_. MiR-30e-5p mimics, NFAT5 siRNA, and DARS2 siRNA were transfected into cells using GenMute siRNA transfection reagent (SignaGen Laboratories, USA). The NFAT5 plasmid was transfected using a Lipojet in vitro siRNA and DNA transfection kit (SignaGen Laboratories, USA). Subsequent experiments were performed 48 h after transfection. Cells were treated with the c-MYC specific inhibitor 10,058-F4 (Selleckchem, USA) at a concentration of 50 mM for 48 h.

### Real-time quantitative PCR

RNA was extracted from tissues and cells by TRIzol reagent (Biosharp, China). The concentration and quality of the total RNA were examined using a Nanodrop 2000 Spectrophotometer. The concentration was between 800 ng/μl and 2500 ng/μl, with the A260/280 between 1.8 and 2.0. MicroRNA reverse transcription was performed using a miRNA cDNA Synthesis Kit with Poly (A) Polymerase Tailing (abm, Canada). The mRNA was reverse-transcribed with PrimeScript RT Master Mix (Takara, Japan). The miR-30e-5p relative expression in HCC tissue was calculated as log2 (2^-ΔΔCT^) based on the threshold cycle and normalized to U6 expression. The mRNA relative expression was calculated as 2^-ΔΔCT^ based on the threshold cycle and normalized to β-actin expression. RT-qPCR was performed on a Bio-Rad iQ5 instrument using SYBR green mix (Toyobo, Japan).

### Protein extraction and western blot analysis

Total protein was extracted by lysis cells in RIPA and 1% PMSF for 30 min on ice, and the lysates were then centrifuged at 12,000 rpm and 4 °C for 15 min. The supernatants were collected and boiled at 95 °C for 5 mins with 5X SDS loading buffer. The total protein quantity was measured by a BCA assay. An appropriate volume of each sample was loaded onto an SDS-PAGE gel. After SDS-PAGE, the proteins were transferred to a PVDF membrane. The PVDF membrane was incubated in blocking buffer (5% skim milk in TBS-T) for 2 h at RT. The membrane was then incubated with a primary antibody at 4 °C overnight. The next day, the membrane was washed with TBS-T and then incubated with a secondary antibody at 4 °C for 1–2 h. Finally, the PVDF membrane was rinsed, and the signals were developed using ECL methods.

### ChIP-PCR and ChIP-seq

The ChIP assay was performed using the Magna ChIP-Seq™ Chromatin Immunoprecipitation and Next Generation Sequencing Library Preparation Kit (17–1010, Millipore, USA). Next-generation sequencing was performed by Orbiotech (China). The ChIP-PCR primers were as follows: NFAT5 promoter forwwad primer 5′- CCCAACCCTAGCACTCCAA-3′ and NFAT5 promoter reverse primer 5′- ATATTGATGCCAGTAGCCA CG-3′. The antibodies for ChIP were as follows: NFAT5 antibody (HPA069711, Sigma-Aldrich, USA) and c-MYC antibody (ChIP Grade, ab32, Abcam, USA).

### Luciferase reporter assay and plasmid construction

A 2000-bp promoter construct of the NFAT5 gene, corresponding to the sequence from nt −2000 to 0 (relative to the transcriptional start site) of the 5′-flanking region of the human NFAT5 gene, was generated from human genomic DNA by PCR. The PCR product was cloned into the Kpnl and Nhel sites of the pGL3-Basic vector. Efficiency of construct was confirmed by DNA sequencing. The 5-flanking deletion constructs of the NFAT5 promoters, NFAT5-promoter-(50 bp, 237 bp, 409 bp, 741 bp, 1012 bp, 1531 bp) were similarly generated by PCR, using the NFAT5-promoter-2000 bp construct as a template. The pBlue-HBV plasmids were transfected into Huh 7 cells. MiR-30e-5p mimics and negative control mimics were transfected into Hep3B cells. The wild-type and mutant MAP4K4 3’UTR sequences are displayed in Supplimentary data. The MAP4K4 3’UTR or NFAT5 promoter was amplified and cloned into the pGL3-reporter vector containing the firefly luciferase gene. Co-transfection is proceede with firefly luciferase report gene, mimics or pBlue-HBV and pRL-TK plasmid with Renilla luciferase gene. The firefly and Renilla luciferase activities were detected 48 h after transfection. The firefly luciferase activities were normalized to Renilla luciferase activities.

### Electrophoresis mobility shift assay (EMSA)

EMSA was performed using a nonradioactive EMSA kit (PIERCE, cat:89,880), The 5′ end of the oligonucleotides was biotin-labeled. Probe was labeled and purified at first, The sample binding reaction system:ddH2O Water 5.8ul, 10X binding buffer 1.5ul, polydI:dC 2ul, sample 4.7ul, Bio-AP1 probes 1ul. The Super-shift reaction system: ddH2O Water 5.8ul, 10X binding buffer 1.5ul, polydI:dC 2ul, sample 4.7ul, Bio-AP1 probes 1ul, the AP1 specific antibody 4.0ul.The reaction procedure was accorded to the manufacturer’s instructions. Using an Imager apparatus (Alpha Innotech, San Leandro, CA) to obtain images.

### Quantitative Methylation changes of NFAT5 promoter

Genomic DNA was isolated from all samples using the Cell genomic DNA extraction Kit (Generay, cat: GK 0122). An Epitect bisulfite kit (Qiagen AG, Basel, Switzerland) was used to perform bisulfite conversion of the genomic DNA. The shrimp alkaline phosphatase (Sequenom, San Diego) was used to remove unincorporated dinucleotide triphosphates.2ul of PCR product was used as a template for the transcription reaction, which was performed by the following PCR. Then T cut/RNase A digestive response procedures was performed. Resin Purification before performing MALDI-TOF MS analysis.The RNase A-treated product was robotically dispensed onto a silicon matrix of preloaded chips (SpectroCHIP; Sequenom, San Diego). The EpiTYPER software version 4.0 was used to detect the methylation ratios of the spectra.

### Immunohistochemical staining (IHC)

IHC for NFAT5 and DARS2 was performed on HCC and para-tumor tissues of patients at Zhongnan Hospital of Wuhan University. The antibodies for IHC were as follows: NFAT5 antibody (HPA069711, Sigma-Aldrich, USA) and DARS2 antibody (ab154606, Abcam, USA).

### Flow cytometry assay for apoptosis and cell cycle

Cells for the FCM apoptosis assay were stained with Annexin-V FITC/PI. FCM was performed 48 h after transfection. The apoptotic rate was calculated as the total sum of the acute apoptotic rate and the terminal apoptotic rate. Cells for the FCM cell cycle assay were stained with PI. FCM was performed 48 h after transfection. Cell counts in each phase of the cell cycle are displayed along with the proportion of the total cell counts.

### Statistical analysis

A paired t-test was performed to assess the variance differences between miR-30e-5p and DARS2 expression in tumor and non-tumor tissues for statistical significance. An unpaired t-test was performed to measure the significance of continuous data. A chi-square test was used to determine the relationship between the clinical data and DARS2 expression. ROC curve generation, Kaplan-Meier survival analysis and COX regression analysis were performed by SPSS 21. Statistical significance was indicated by *P* < 0.05 (*), *P* < 0.01 (**), and *P* < 0.0001 (***). All experiments were repeated in triplicate.

## Results

### NFAT5 is downregulated in HCC, decelerating cell cycle progression and inducing apoptosis

**Fig. 1:**
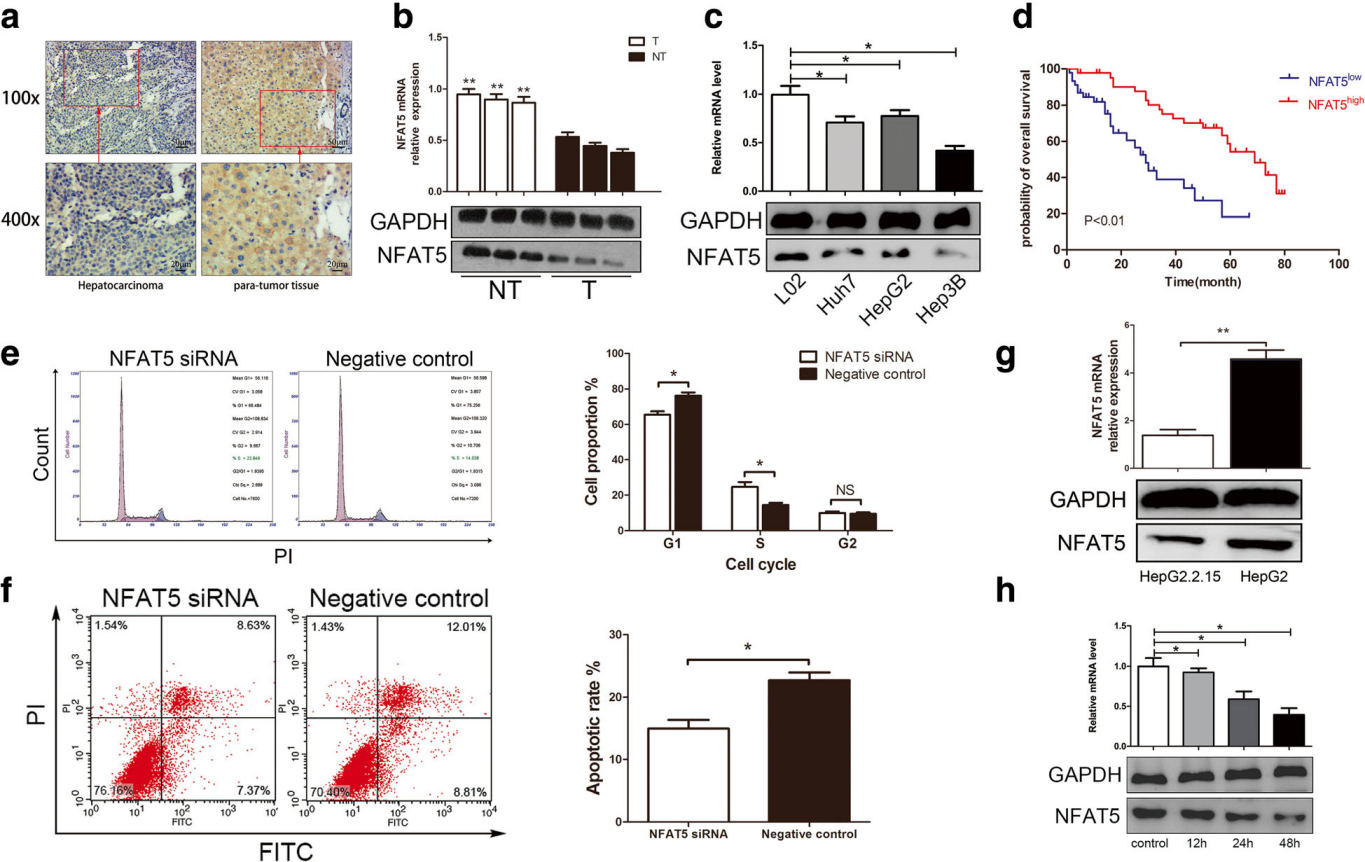
NFAT5 was identified as a tumor suppressor in HCC. **a** Immunohistochemical analyses of HCC and para-tumor tissues showed that NFAT5 was downregulated in HCC tissues and expressed in the cytoplasm. Images at 100X and 400X magnification are displayed. **b** NFAT5 mRNA and protein was detected by RT-qPCR and western blot assay from 3 HCC patients. NFAT5 mRNA and protein was downregulated in HCC tumor tissue. **P < 0.01. **(C)** NFAT5 mRNA and protein expression in different HCC cell lines, compared to normal liver cell L02. All HCC cell produced less NFAT5 than L02. *P < 0.05. **d** Kaplan-Meier survival analysis for NFAT5. HCC patients with high NFAT5 expression have longer survival. **e** Left panel: FCM cell cycle assay showed NFAT5 knockdown accelerated S phase entry of HCC cells. Right panel: Statistical analysis of cell proportion in each cell cycle phase. *P < 0.05. **f** Left panel: NFAT5 knockdown suppressed the cell apoptosis, evaluated by FCM. Right panel: Statistical analysis showed NFAT5 knockdown significantly reduced apoptotic rate of HCC. *P < 0.05. **g** NFAT5 mRNA and protein expressed less in HepG2.2.15 than HepG2, detected by RT-qPCR and western blot. *P* **=** 0.002. **h** NFAT5 mRNA and protein expression was measured by RT-qPCR and Western-blotting in Huh 7 cells transfected with the plasmid pBlue-HBV at different times

The expression of NFAT5 was examined via immunohistochemical staining of 90 pairs of liver tumor tissues/corresponding para-tumor tissues using antibodies against NFAT5. The results showed that NFAT5 expression was reduced in liver tumor tissues compared with expression in para-tumor tissues (Fig. 1a). High-magnification images showed that NFAT5 was mainly located in the cytoplasm. Besides, NFAT5 expressed lower in HBV-associated HCC (Additional file 2: Figure S1A). To further confirm the expression of NFAT5, three liver tumor tissues and corresponding para-tumor tissues were selected for mRNA and Western blot analyses. The results indicated that the mRNA and protein expression of NFAT5 was low in liver tumor tissues, but high in corresponding para-tumor tissues (Fig. 1b). The expression of NFAT5 was also significantly downregulated in various hepatoma line cells compared with expression in L02 cells (Fig. 1c). The potential correlation between the expression status of NFAT5 and clinical pathologic parameters of patients with HCC was further analyzed (Additional file 1: Table S1). The results indicated that aberrant expression of NFAT5 was negatively correlated with the histologic grade (*P* = 0.035), HBV infection (*P* < 0.01) and the Barcelona Clinic Liver Cancer (BCLC) stage (*P* < 0.01).

Next, we performed a Kaplan-Meier analysis to investigate the relationship between NFAT5 expression and the survival of patients with HCC. At the last clinical follow-up, 33 of 90 patients remained alive, while 57 had died, and the median overall survival (OS) of all patients was 29.40 M. The 1-year survival rate was 90%; the 3-year survival rate was 42%; and the 5-year survival rate was 13%. The survival time was longer in the group showing higher expression of NFAT5 (Fig. 1d). The mean OS of patients with low or high NFAT5 expression was 23.83 M (range, 1~ 67 M), and 57.82 M (range, 4~80 M), respectively. There was a significant difference in the median OS of patients with low or high NFAT5 expression (log-rank test χ2 = 27.29, *P* < 0.01). These results demonstrated a clear positive correlation between the levels of NFAT5 protein expression and OS in patients with HBV-associated HCC.

Furthermore, we investigated the effect of NFAT5 on cell cycle and apoptosis in hepatoma cells. Flow cytometry (FCM) cell cycle analysis indicated that knockdown of NFAT5 accelerated S phase entry (Fig. 1e). Moreover, knockdown of NFAT5 resulted in a lower apoptotic rate (Fig. 1f). All of these data indicated NFAT5 is a cancer suppressor gene.

### HBV inhibits NFAT5 expression by inducing hypermethylation of the AP1-binding site in the NFAT5 promoter

**Fig. 2:**
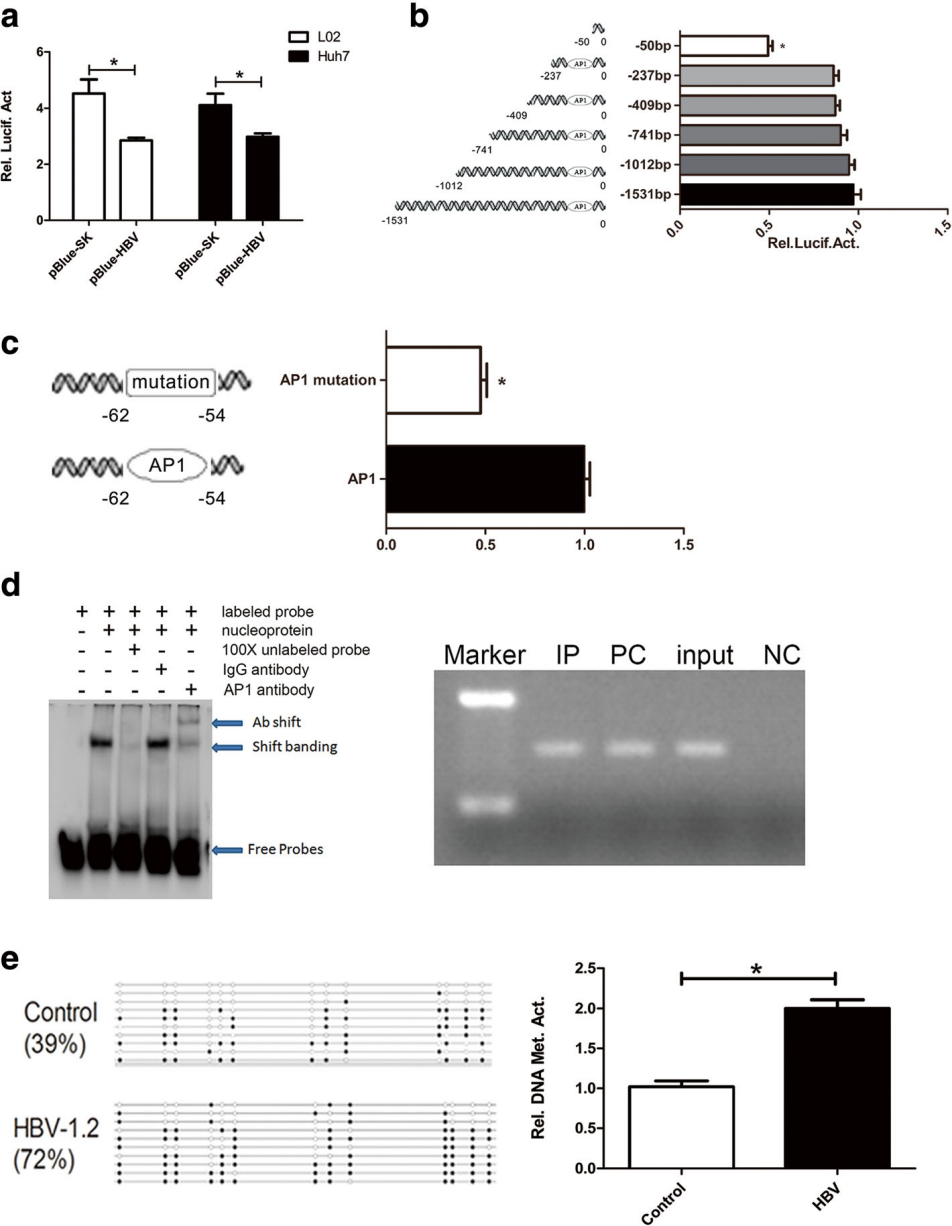
HBV induced hypermethylation of AP1 binding site in NFAT5 promoter for suppressing its expression. **a** Diagrams of reporter constructs containing the luciferase (Luc) gene under the control of the human NFAT5 promoter. L02 and Huh7 cell lines were co-transfected with pBlue-HBV or control plasmid pBlue-SK along with the constructed reporter plasmids with NFAT5 promoters. **b** NFAT5 promoter core region was identified in hepatoma cells by luciferase reporter gene assays. Length of 50 bp expresses a significant decrease of luciferase activity. *P < 0.05 **(c)** AP1 Binding Elements was identified by specific mutation. Mutant AP1 binding site represses luciferase activity. **d** Left panel: Analyses the binding of AP1 to the NFAT5 promoter by EMSA. AP1 probe was generated by annealing single-stranded and end-labeled oligo nucleotides containing the cognate NFAT5 promoter region. 1st lane: negative control reaction (labeled probe). 2nd lane: conventional reaction (including the nucleoprotein of activated target transcription factors with labeled probes). 3rd lane: probe cold competing reaction (including the nucleoprotein of activated target transcription factors, labeled probe and unlabeled probe with 100 times amount of labeled probe). 4th lane: cold competing reaction of mutated probe (including the nucleoprotein of activated target transcription factors, the labeled probe and mutated probe with 100 times amount of labeled probe). 5th lane: Super-shift reaction (including the nucleoprotein of activated target transcription factors, labeled probe and the specific antibody of AP1). Right panel: Determination the binding of AP1 to the NFAT5 promoter by ChIP assays. The lanes from left to right are Marker, experimental group, positive control group, input group, and negative control group. **(E)** Left panel: The methylation status of CpG island of NFAT5 promoter in response to HBV regulation was analyzed by bisulfite sequencing analysis in the Huh7 cells. Right panel: The methylation status of CpG island of NFAT5 promoter in response to HBV regulation was analyzed by MSP analysis as well

To explore the role of HBV in the regulation of NFAT5 expression, we determined the levels of NFAT5 mRNA and protein expression in HepG2 and HepG2.2.15 cells. The results showed that NFAT5 mRNA and protein levels were lower in HepG2.2.15 cells than in HepG2 cells (Fig. 1g). Thus, we considered HBV to be responsible for the inhibition of NFAT5 gene expression observed in HepG2.2.15 cells. This hypothesis was supported by measuring NFAT5 mRNA and protein expression levels at different time points after transfection with plasmids (pBlue-HBV) containing 1.3-fold HBV genomes in Huh 7 cells (Fig. 1h). Next, a luciferase activity assays illustrated that NFAT5 promoter activity was significantly decreased by the presence of HBV in Huh7 and L02 cells (Fig. 2a).

We generated Huh7 cells transfected with plasmids containing the luciferase reporter gene under the control of wild-type and truncated NFAT5 promoters. The results of indicated that the sequence between −237 bp and −50 bp is critical for the activation of NFAT5 (Fig. 2b). Analyses of the cis-regulatory elements contained in the sequence from −237 bp to −50 bp in the NFAT5 promoter revealed that there is one AP1-binding site in this region. Therefore, we altered its sequence through site-directed mutagenesis and found that mutation of the AP1-binding site significantly decreased NFAT5 promoter activity (Fig. 2c). These results suggest that AP1 recognition elements are required to activate NFAT5 expression. For confirming that the AP1-binding site is a key transcriptional regulatory region of the NFAT5 gene, we performed EMSA. The supershift strips confirmed that the AP1-specific antibody was capable of combining with the AP1-binding site in the NFAT5 promoter (Fig. 2d). Chromatin fragments were prepared from Huh7 cells and immunoprecipitated with antibodies against AP1. The locations of the PCR products are indicated as Chip under the simplified genomic structures of the *NFAT5* promoter (Fig. 2d). Overall, we conclude AP1 binding element is required for NFAT5 transcription.

Next we studied the epigenetic mechanisms through which HBV inhibits NFAT5 via bisulfite-sequencing PCR and MSP. We identified that the core functional sequence of the AP1 element was located in the region from −62 bp to −54 bp (GTGCCGCC) (Additional file 2: Figure S1B), which is within the second CpG island of the NFAT5 promoter. We then examined the DNA methylation pattern at the CpG islands of the NFAT5 promoter, inferring that the degree of methylation within the NFAT5 promoter was 72% in Hep3B cells transfected with the pCMV-HBV-1.3 plasmid, while it was 39% in Hep3B cells transfected with an empty plasmid (Fig. 2e). We then assessed the expression level of NFAT5 in Hep3B cells that were transfected with the pCMV-HBV-1.3 plasmid and then treated with 5 μM Aza (a DNA methylation inhibitor) for 72 h. The results showed the mRNA expression level of NFAT5 and the luciferase activity associated with the NFAT5 promoter were significantly decreased in Hep3B cells transfected with the pCMV-HBV-1.3 plasmid, whereas both were increased in a concentration-dependent manner when the cells were treated with 5-Aza-2′ deoxycytidine (Additional file 2: Figure S1C). Thus, we conclude that HBV downregulates the expression of NFAT5 in hepatoma cells by inducing DNA hypermethylation at the NFAT5 promoter.

### HBV inhibits NFAT5 expression via inhibiting miR-30e-5p

**Fig. 3:**
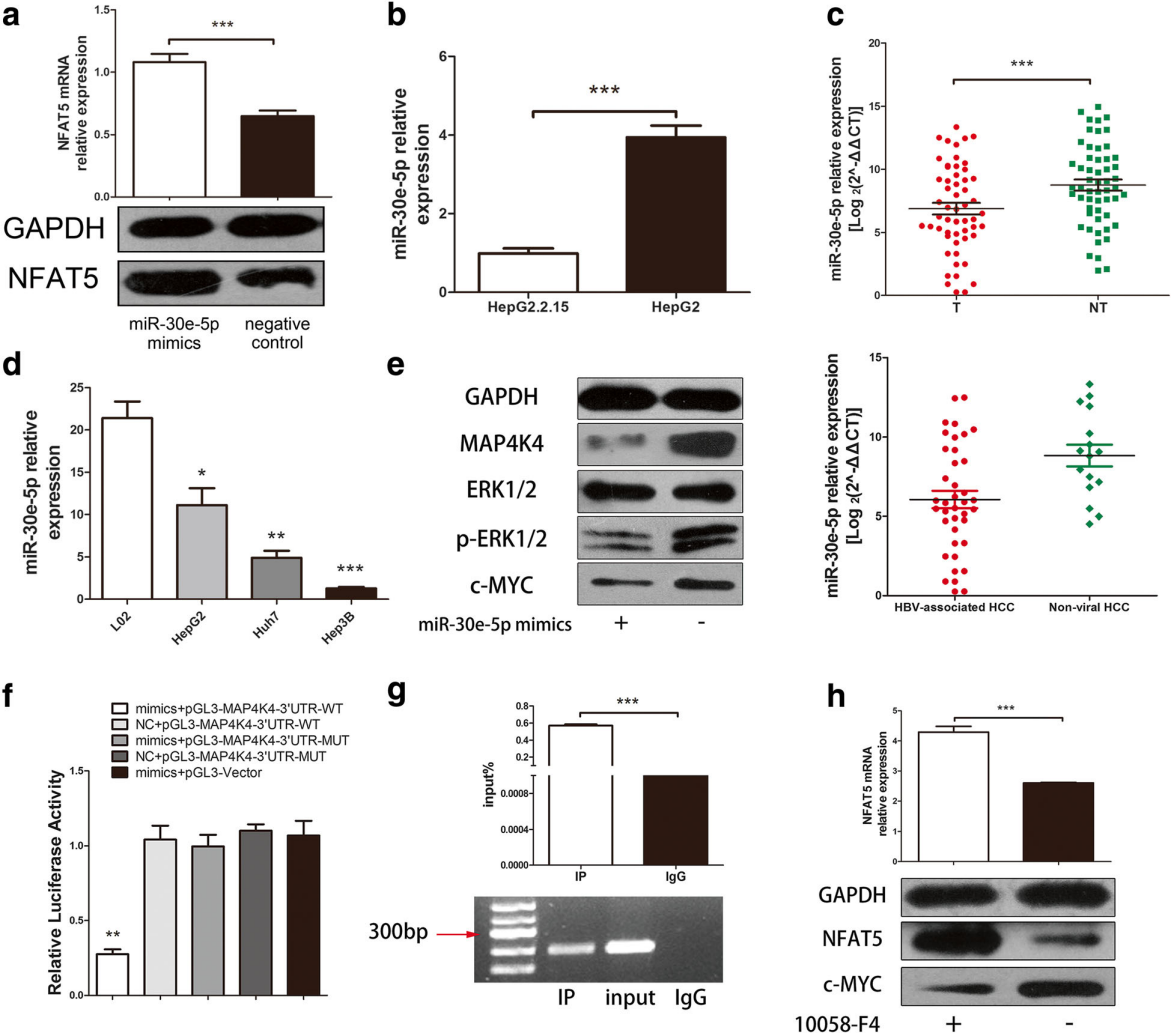
HBV downregulated NFAT5 via inhibiting miR-30e-5p and activating MAPK signaling pathway. **a** MiR-30e-5p overexpression induced NFAT5 mRNA and protein expression in Hep3B, as verified by RT-qPCR and western blot analysis. **b** MiR-30e-5p relative expression was lower in HepG2.2.15 than HepG2 (*P* = 0.0008). **(C)** Top panel: MiR-30e-5p was downregulated in HCC tissues (*P* < 0.0001), as determined by RT-qPCR in 55 pairs of HCC tissues and para-tumor tissues. The relative expression of miR-30e-5p was calculated by log2 (2^-ΔΔCT) and normalized to U6 expression. Botton panel: MiR-30e-5p was downregulated in HBV-associated HCC (*n* = 39), compared to non-viral HCC (*n* = 19). *P* = 0.0056. **d** MiR-30e-5p expression was downregulated in the HCC cell lines HepG2 (*P* = 0.0217), Huh7 (*P* = 0.0015) and Hep3B (P < 0.0001) compared with that in the normal liver cell line L02. **e** Western blot analyses revealed that MAP4K4, p-ERK1/2, and c-MYC expression decreased when cells were transfected with miR-30e-5p mimics. **f** A luciferase reporter assay showed that co-transfection with miR-30e-5p mimics and the wild-type MAP4K4 3’UTR decreased relative luciferase activity (P = 0.0015). WT = wild-type, MUT = mutant, mimics = miR-30e-5p mimics, vector = empty vector. **g** ChIP-PCR revealed that the c-MYC protein directly interacted with DNA sequence fragments containing the NFAT5 promoter. The picture displays the AGE result after ChIP-PCR. IP = immunoprecipitation group (with the c-MYC antibody), input = group without any antibody as a positive control, IgG = group with IgG as a negative control. Quantitative analysis of AGE showed that IP group was significantly higher than the IgG group (*P* < 0.0001). **h** RT-qPCR and western blot showed that HCC cells treated with the c-MYC inhibitor 10,058-F4 expressed higher levels of NFAT5 mRNA (*P* < 0.0001) and protein. The efficiency of 10,058-F4 was verified by a western blot analysis of c-MYC

Because we found that HBV induces inhibition of NFAT5 by AP1, we were interested in other pathway of HBV in modulating NFAT5 expression. We screened several miRNA regulators of NFAT5 according to a literature review. We found that upregulation of miR-30e-5p positively mediated NFAT5 expression at both the mRNA and protein levels (Fig. 3a). Additionally, miR-30e-5p expression was reduced in HepG2.2.15 cells compared with that in HepG2 cells, indicating that HBV could suppress miR-30e-5p expression (Fig. 3b). To investigate the role of miR-30e-5p in HBV-associated HCC, we evaluated miR-30e-5p expression in 55 HCC patients, and the results showed that miR-30e-5p expression was lower in HBV-associated HCC tissues than para-tumor tissues (Fig. 3c and Additional file 2: Figure S1D). Furthermore, miR-30e-5p was downregulated in HBV-associated HCC compared with non-viral HCC (Fig. 3c) and was also downregulated in HCC cell lines compared with the normal liver cell line L02 (Fig. 3d). Additionally, we detected HBsAg and HBeAg in the medium of HepG2.2.15 cells transfected with miR-30e-5p mimics, via electrochemiluminescence immunoassays, and the results demonstrated that the levels of both HBsAg and HBeAg decreased when miR-30e-5p was overexpressed (Additional file 2: Figure S1E). In addition, miR-30e-5p mimics promoted NFAT5 expression and suppress HBx expression in Hep3B and HepG2.2.15 cells carrying an integrated fragment of HBV genomic DNA in their chromosomes (Additional file 2: Figure S1F). This observation might indicate that HBV and miR-30e-5p mutually regulate each other. Taken together, the results suggest that HBV indirectly suppresses NFAT5 by regulating miR-30e-5p expression.

### miR-30e-5p induces NFAT5 expression by suppressing the MAP4K4 signaling pathway

NFAT5 is not a target gene of miR-30e-5p according to prediction using TargetScan. A previous study showed that the MAPK signaling pathway could regulate the activation of NFAT5 [18]. We identified potential binding sites and target genes of miR-30e-5p and chose the MAP4K4 signaling pathway for further study. A miR-30e-5p-binding site is located at positions 3128–3135 of the MAP4K4 3’UTR (Additional file 2: Figure S1G). KEGG (Kyoto Encyclopedia of Genes and Genomes, http://www.kegg.jp/) analysis indicated that MAP4K4 is involved in the MAPK signaling pathway by mediating the phosphorylation of MEKK1 to phosphorylate ERK1/2 and ultimately induce the translation of c-MYC (Additional file 2: Figure S1I). Hence, we studied the effect of miR-30e-5p overexpression on the MAPK signaling pathway, and the results showed that miR-30e-5p inhibited MAP4K4 expression and thereby suppressed the downstream protein c-MYC by decreasing the level of phosphorylated ERK1/2 in Hep3B cells (Fig. 3e). A luciferase reporter assay was performed to verify that MAP4K4 is the direct target gene of miR-30e-5p, in which we mutated the miR-30e-5p-binding site in the MAP4K4 3’UTR (Additional file 2: Figure S1H). The results showed that only the wild-type 3’UTR could interact with miR-30e-5p mimics, which attenuated luciferase activity, whereas miR-30e-5p failed to repress luciferase activity when the binding sequence was mutated (Fig. 3f). Together, these data revealed that miR-30e-5p can suppress the MAPK signaling pathway through directly targeting MAP4K4.

We next sought to identify the NFAT5 regulator by predicting potential proteins binding to the NFAT5 promoter. We defined 2000 bp upstream from the transcriptional starting site as the NFAT5 promoter. We entered the NFAT5 promoter sequence into ALGGEN PROMO, provided by the Polytechnic University of Catalunya (http://alggen.lsi.upc.es/). This program identified numerous proteins that could bind to the NFAT5 promoter, including c-MYC. The c-MYC-binding site is located at positions −1369 bp~ − 1364 bp of the NFAT5 promoter, with the sequence CACGTG (Additional file 2: Figure S1J). We then performed a ChIP assay to confirm this prediction. We used a c-MYC antibody to pull down sheared, crosslinked chromatin and determined whether the NFAT5 promoter was included in the pulled-down DNA. ChIP-PCR primers were designed to amplify the region of the NFAT5 promoter that contained the c-MYC-binding site. The sample incubated with the c-MYC antibody (IP group) was amplified via PCR, while the sample incubated with IgG (as a negative control) was not amplified (Fig. 3g). We quantitatively analyzed the ChIP results by measuring the signal of the PCR-amplified bands from the IP and IgG groups and then calculating the ratio of these signals to the input signal. The IP group exhibited a significantly stronger signal than the IgG group (Fig. 3g). We next treated the Hep3B HCC cell line with 10,058-F4, a c-MYC inhibitor, and the results showed that NFAT5 expression was increased at both the mRNA and protein levels after 48 h of treatment (Fig. 3h), suggesting that c-MYC inhibited NFAT5 expression by binding to the NFAT5 promoter. Based on the data obtained thus far, it is reasonable to conclude that miR-30e-5p induces NFAT5 expression by suppressing the MAP4K4 signaling pathway.

### NFAT5 inhibits HCC tumorigenesis by negatively mediating the target gene DARS2

**Fig. 4:**
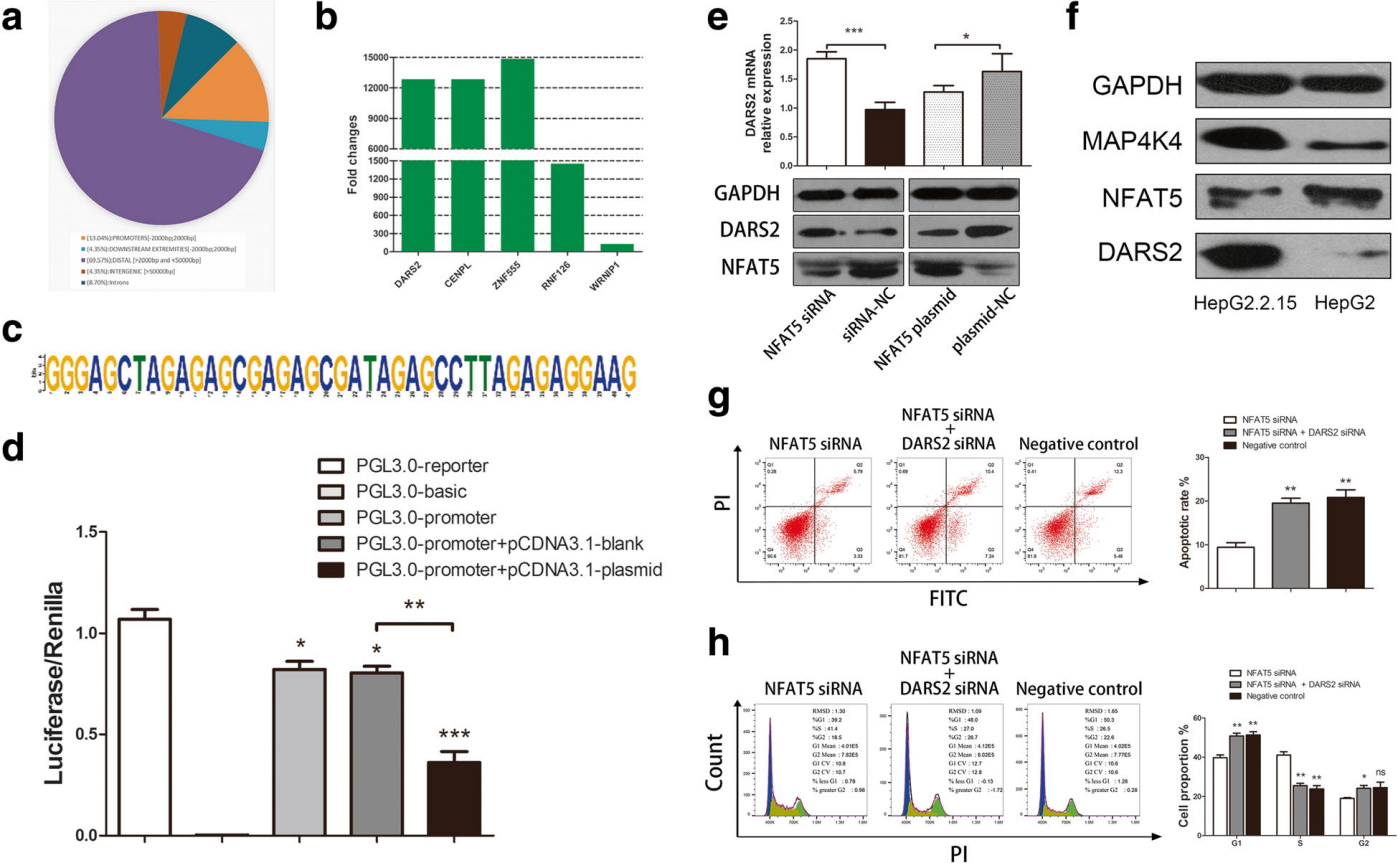
ChIP-seq revealed that NFAT5 negatively mediated DARS2 to inhibit HCC tumorigenesis. **a** Proportion of peak annotation detected by ChIP-seq. **b** Fold change of novel NFAT5 targets. Fold change was defined as the ratio of peak reads in the IP group to those in the IgG group. **c** Motif sequence of peaks bound by the NFAT5 protein. The motif was highly conserved. **d** Luciferase report assay confirm that NFAT5 bound to DARS2 promoter. Reporter: positive control; basic: negative control; promoter: DARS2 promoter; blank: control plasmid; plasmid: NFAT5 plasmid. **e** RT-qPCR and Western blot analyses demonstrated that NFAT5 negatively mediated DARS2 protein expression. NFAT5 overexpression and knockdown efficiency were verified by western blot analyses of NFAT5. **f** Western blot demonstrated HepG2.2.15 expressed higher MAP4K4, DARS2 and lower NFAT5 protein, which confirmed HBV upregulated DARS2 via miR-30e-5p/MAPK/NFAT5 signaling pathway. **g** Left panel: In an FCM apoptosis assay, NFAT5 knockdown in HepG2 led to an attenuation of apoptosis, while knockdown of DARS2 did not regulate apoptosis. Right panel: Statistical analysis showed that NFAT5 siRNA attenuated the apoptosis rate (*P* = 0.0054 vs negative control), while co-transfection of NFAT5 siRNA and DARS2 siRNA inhibited NFAT5-induced apoptosis (*P* = 0.0032 vs NFAT siRNA, no significant difference compared with negative control). Apoptotic rate = acute apoptotic rate (right lower quadrant) + terminal apoptotic rate (right upper quadrant). **h** Left panel: FCM cell cycle analysis revealed that transfection with NFAT5 siRNA into HepG2 accelerated cell cycle progression, while co-transfection of NFAT5 siRNA and DARS2 siRNA failed to accelerate cell cycle progression. Right panel: Statistical analysis showed that NFAT5 knockdown decreased the proportion of cells in G1 phase (*P* = 0.0059 vs negative control) and increased the proportion of cells in S phase (*P* = 0.0021 vs negative control). Co-transfection of NFAT5 siRNA and DARS2 siRNA failed to accelerate cell cycle progression (*P* = 0.0051 vs NFAT5 siRNA in G1 phase, *P* = 0.0019 vs NFAT5 siRNA in S phase, no significant difference compared with negative control)

To identify the molecular mechanism underlying the effect of NFAT5 on HBV-associated HCC, we further investigated the downstream protein of NFAT5 using ChIP together with next-generation sequencing. The DNA fragments that were bound by NFAT5 and detected via next-generation sequencing were annotated (Fig. 4a). We chose seven genes for screening of the target gene (Additional file 1: Table S2). Two of these targets were noncoding RNAs (DUSP5P and UBE2MP1) and were therefore excluded from further analysis. ZNF555 showed the largest difference (fold change = 14,844), while WRNIP1 showed the smallest (fold change = 125) (Fig. 4b). The common motif sequence of these targets is highly conserved (Fig. 4c). We performed a literature review of the 5 novel targets and found that DARS2 (fold change = 12,831) has not been studied in any tumor disease to date. Several clinical studies have shown that mutations in DARS2 are associated with leukoencephalopathy [19, 20]. We found that DARS2 mRNA and protein expression were negatively regulated by NFAT5 in NFAT5 gene overexpression and knockdown experiments (Fig. 4e). When we examined the ChIP-seq data, we found that the NFAT5-binding DNA fragments of DARS2 were located in the DARS2 promoter (Additional file 1: Table S2), which suggested that NFAT5 suppresses DARS2 by binding to its promoter. This finding was confirmed by luciferase report assay. When we co-transfected pCDNA3.1-NFAT5 plasmid and PGL3.0-DARS2 promoter, the activity of luciferase/renilla was obviously suppressed (Fig. 4d). It inferred NFAT5 indeed inhibited DARS2 by binding to its promoter. We next investigated the effect of HBV on NFAT5 and DARS expression using HepG2.2.15 cells carrying an integrated fragment of HBV genomic DNA as well as HepG2 cells not carrying HBV genomic DNA. The results showed that HBV upregulated DARS2 expression and inhibited NFAT5 expression simultaneously (Fig. 4f). Therefore, it is reasonable to suggest that the role of NFAT5 in the development of HCC is realized by suppressing DARS2. Hence, we investigated whether the effect of NFAT5 on cell apoptosis and the cell cycle would be hindered by altering DARS2 expression in hepatoma cells to verify our hypothesis. We found that knockdown of NFAT5 reduced the rate of apoptosis, whereas co-transfection of NFAT5 siRNA and DARS2 siRNA had little effect on cell apoptosis in HepG2 cells (Fig. 4g). This result indicated that the apoptosis-promoting effect of NFAT5 on hepatoma cells was inhibited by DARS2. We also studied the effects of NFAT5 and DARS2 on the cell cycle in HepG2 cells. FCM revealed that NFAT5 knockdown contributed to cell cycle progression and, similarly, to apoptosis, whereas co-transfection of NFAT5 siRNA and DARS2 siRNA did not affect the cell cycle (Fig. 4h). We calculated the number of cells in each phase of the cell cycle and determined that NFAT5 knockdown resulted in an acceleration of S phase (Fig. 4h). These results suggested that NFAT5 inhibits HCC progression by suppressing DARS2 expression.

### DARS2 is strongly upregulated in HCC and is associated with HCC progression

**Fig. 5:**
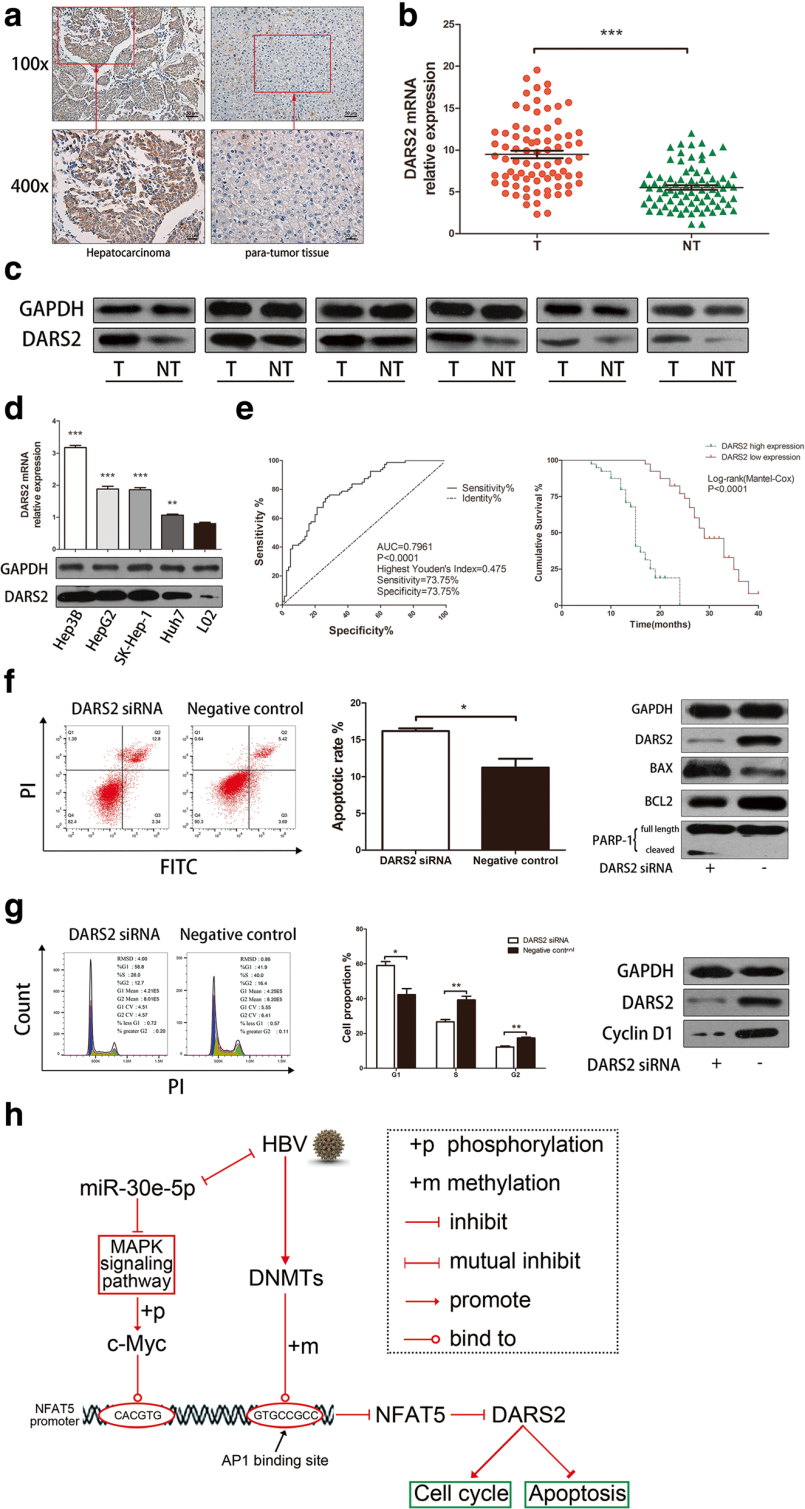
DARS2 was upregulated in HCC and promoted cell cycle progression and inhibited apoptosis in HCC. **a** Immunohistochemistry for DARS2 in HCC tissues and para-tumor tissues. DARS2 was upregulated in HCC and expressed in the cytoplasm. Images at 100X and 400X magnification are shown. **b** DARS2 mRNA expression was examined in 80 pairs of HCC tissues and para-tumor tissues. DARS2 relative expression was calculated by 2^-ΔΔCT^, normalized to β-actin expression. DARS2 was upregulated in HCC tissues compared with para-tumor tissues (*P* < 0.0001). **c** DARS2 protein expression was also higher in HCC tissues than in para-tumor tissues. DARS2 protein expression, detected by western blotting, in six pairs of tissues is displayed. **d** DARS2 mRNA and protein expression in the HCC cell lines Hep3B (*P* < 0.0001), HepG2 (*P* < 0.0001), SK-Hep-1 (*P* < 0.0001) and Huh7 (*P* = 0.0021) compared with the normal liver cell line L02. Hep3B cells expressed the highest level of DARS2. **e** Left panel: An ROC curve was drawn based on DARS2 mRNA expression in 80 patients. Youden’s index = sensitivity% + specificity%-1. The cutoff value, sensitivity and specificity were determined by the highest Youden’s index, which is shown in the figure. Right panel: A Kaplan-Meier analysis was used to determine the correlation between DARS2 expression and survival. Follow-up visits occurred for 40 months after the patients were initially diagnosed with HCC. Patients with higher DARS2 expression had a shorter survival time. **f** Left panel: Apoptosis was analyzed by FCM in Hep3B cells transfected with DARS2 siRNA for 48 h. The apoptosis of cells transfected with DARS2 siRNA was higher than those transfected with a negative control. Middle panel: Statistical analysis of the FCM apoptosis assay. The apoptotic rate increased when Hep3B cells were transfected with DARS2 siRNA for 48 h. Right panel: Biomarkers for apoptosis, including BAX, BCL-2 and cleaved PARP-1, were detected by western blot analyses. DARS2 knockdown induced BAX and cleaved PARP-1 expression and reduced BCL-2 expression. **g** Left panel: The cell cycle was analyzed by FCM in Hep3B cells transfected with DARS2 siRNA for 48 h. DARS2 knockdown inhibited cell cycle progression. Middle panel: Statistical analysis of the FCM cell cycle assay. DARS2 knockdown induced an arrest prior to S phase. The proportion of cells in G1 phase increased (*P* = 0.0151), and the proportion of cells in S phase decreased (*P* = 0.0072). Right panel: Cyclin D1 protein decreased after 48 h of DARS2 knockdown, as detected by western blot. The efficiency of DARS2 knockdown was verified by a western blot analysis of DARS2. **h** The whole pathway of this research. HBV stimulated DNMTs for inducing the hypermethylation of AP1 binding site on NFAT5 promoter (−54 bp~ − 62 bp), for inhibiting NFAT5 transcription. On the other hand, HBV activated MAPK signaling pathway by suppressing miR-30e-5p, in order to inhibit NFAT5 indirectly. All in all, HBV is able to upregulated DARS2 by inhibiting NFAT5. DARS2 is an oncogene, associated with HCC cell apoptosis and cell cycle progression

To study the role of DARS2 in HCC progression, we measured DARS2 expression in 80 HCC patients who voluntarily provided samples. The result showed that the more DARS2 protein were stained in tumor tissues than in non-tumor tissues in immunohistochemistry assays, suggesting that DARS2 is upregulated in HCC tissues (Fig. 5a). DARS2 mainly localized to the cytoplasm in HCC. Furthermore, we found that DARS2 mRNA and protein expression was higher in tumor tissues than in non-tumor tissues (Fig. 5b and c). Fifteen pairs of tissues showing a favorable representation are shown (Additional file 2: Figure S1K). We next examined DARS2 expression in hepatoma cell lines, including Hep3B, HepG2, SK-Hep-1 and Huh7 cells, and the human embryonic liver cell line L02. The results indicated that DARS2 mRNA and protein expression was higher in hepatoma cell lines than in L02 cells (Fig. 5d). Hep3B cells exhibited the highest expression among the examined hepatoma cell lines. Thus, we decided to use Hep3B cells as the cell model for the subsequent experiments.

We further investigated the correlation between DARS2 and clinical data through analyzing DARS2 expression in 80 HCC patients along with their clinical characteristics. We first generated an ROC curve according to the observed DARS2 mRNA expression levels (Fig. 5e). The area under the ROC curve (AUC) was 0.7961 (*P* < 0.0001). The highest Youden’s index was 0.475 when the value of cutoff was 6.589, the sensitivity and specificity for the DARS2 gene were both 73.75%. Furthermore, we collected the clinical data about HCC patient in our hospital, and found that high expression of DARS2 was related to tumor size (χ2 = 6.084, *P* = 0.014), HBV infection (χ2 = 24.757, P < 0.0001), BCLC stage (χ2 = 15.245, *P* = 0.002), cell differentiation (χ2 = 22.667, P < 0.0001), distal metastasis (χ2 = 5.165, *P* = 0.023), and portal vein invasion (χ2 = 7.813, *P* = 0.005) using a chi-square test (Table 1). Based on this analysis, we examined DARS2 expression in patients with different characteristics. Patients with a tumor diameter greater than 2 cm exhibited lower DARS2 expression (Additional file 3: Figure S2A). DARS2 was upregulated in HBV-infected patients (Additional file 3: Figure S2B). In addition, the increase in DARS2 expression was correlated with BCLC stage (stage 0-A to stage D) (Additional file 3: Figure S2C) and differentiation status (well to poor) (Additional file 3: Figure S2D). Moreover, DARS2 was upregulated in patients with distal metastasis (Additional file 3: Figure S2E) and portal vein invasion (Additional file 3: Figure S2F). Additionally, we performed a Kaplan-Meier and log-rank survival analysis based on DARS2 expression. Patients with high DARS2 expression exhibited a shorter survival time than patients with low DARS2 expression (Fig. 5e). A univariate Cox regression analysis indicated that DARS2 expression was associated with disease-specific survival as well as other characteristics, such as BCLC stage, differentiation, and portal vein invasion. A multivariate Cox regression analysis was then performed, which revealed tight correlations between the survival time of patients and DARS2 expression, BCLC stage, and cell differentiation (Additional file 1: Table S3). These results indicated that DARS2 strongly contributes to HCC development, showing great potential to be used as a diagnostic marker and a therapeutic target in the future.

**Table 1:** Correlation between clinical pathological characteristics of HCC patients and DARS2 expression

Clinical pathological	*n*	DARS2	Proportion	χ^2^	*P* value
characteristics		high expression, *n*	(%)		
Gender				0.721	0.396
Male	74	36	48.65		
Female	6	4	66.67		
Age (years)				0.053	0.818
≥60	31	16	51.61		
<60	49	24	48.98		
Tumor size (cm)				6.084	0.014
≥2	43	27	62.79		
<2	37	13	35.14		
HBV infected				24.757	<0.0001
Yes	46	34	73.91		
No	34	6	17.65		
BCLC stages				15.245	0.002
0-A	21	6	28.57		
B	23	8	34.78		
C	21	13	61.90		
D	15	13	86.67		
Differentiation				22.667	<0.0001
Well	30	7	23.33		
Moderate	20	8	40.00		
Poor	30	25	83.33		
Liver cirrhosis				0.061	0.805
Yes	57	28	49.12		
No	23	12	52.17		
Intrahepatic metastasis				0.065	0.799
Yes	59	29	49.15		
No	21	11	52.38		
Distal metastasis				5.165	0.023
Yes	11	9	81.82		
No	69	31	44.93		
Portal vein invasion				7.813	0.005
Yes	21	16	76.19		
No	59	24	40.68		
AFP (μg/L)				2.515	0.284
≤25	8	3	37.50		
25–400	27	11	40.74		
>400	45	26	57.78		

### DARS2 promotes HCC tumorigenesis by accelerating cell cycle progression and attenuating cell apoptosis

To elucidate the molecular mechanism underlying effect of DARS2 on HCC progression, we investigated the effect of DARS2 on cell apoptosis and the cell cycle in hepatoma cells. Three siRNAs were designed to knock down DARS2, and the first (siRNA#1), which exhibited the greatest knockdown effect, was used for the following experiments (data not shown). The results of apoptosis assays of Hep3B cells stained with Annexin-V FITC and PI were detected using a flow cytometer after transfection with DARS2 siRNA for 48 h and showed that the knockdown of DARS2 resulted in a higher apoptotic rate than in the negative control (Fig. 5f). Several important biomarkers of apoptosis were then examined by Western blot analyses. Knockdown of DARS2 increased BAX expression and inhibited BCL2 expression, leading to augmentation of the BAX/BCL2 ratio, and cleaved PARP-1 expression was also increased (Fig. 5f). These results implied that DARS2 attenuated cell apoptosis. We further explored whether DARS2 might have an effect on cell cycle progression. Hep3B cells were transfected for 48 h and then stained with PI. FCM showed that knockdown of DARS2 caused more cells to arrest in G1 phase and fewer cells to enter S phase and G2 phase (Fig. 5g). Based on these results, we next examined the expression of cyclin D1, a critical factor in cell cycle progression, after knockdown of DARS2 in Hep3B cells. The results showed that cyclin D1 protein expression was reduced after knockdown of DARS2 compared with expression in the negative control group (Fig. 5g), which indicated that DARS2 accelerated cell cycle progression in hepatoma cells. Together, the above data indicate that HBV suppresses NFAT5 expression by inducing hypermethylation of the AP1-binding site in the NFAT5 promoter, in addition inhibiting NFAT5 through miR-30e-5p targeting of the MAP4K4 signaling pathway, and the inhibition of NFAT5 enhances HCC tumorigenesis by promoting the expression of DARS2 as a downstream target gene (Fig. 5h).

## Discussion

HBV is the most common hepatitis virus and causes chronic infections in the human liver. Recent findings suggest that osmotic stress plays an important role in inflammation and tumorigenesis [2]. NFAT5/TonEBP/OREBP is the only known osmotic pressure-sensitive transcription factor in mammals and plays a vital role in enhancing cell survival, migration and proliferation, vascular remodeling, tumor invasion and angiogenesis [21]. NFAT5 has been shown to be involved in the pathogenesis of multiple cancers. Knocking out NFAT5 reduces the invasiveness of melanoma, and the tumor suppressor gene miR-211 is thought to downregulate the expression of NFAT5 in melanoma [22]. NFAT5 is located at a central node that promotes metastasis in melanoma [12]. Some studies on breast and colon cancer have confirmed that the α6β4 integrin cluster promotes the expression of NFAT5 and the invasiveness and migratory capabilities of tumor cells [23]. NFAT5 may therefore promote tumor cell migration, leading to the formation of various new tumors.

Here, we present the first evidence showing that NFAT5 is involved in the proliferation of HBV-associated HCC. However, we found that NFAT5 has a completely different function in HCC than in other types of human cancers, including breast cancer, colon carcinoma, lung adenocarcinoma, renal cell carcinoma and melanoma [2, 3, 16, 22, 24]. The key finding of the current study is that NFAT5 acts as a tumor suppressor by inhibiting cell cycle progression and promoting tumor cell apoptosis in vitro. We demonstrated the presence of a clearly positive correlation between the level of NFAT5 protein expression and OS in patients with HBV-associated HCC, suggesting that NFAT5 is a cancer suppressor gene. We further investigated the mechanism whereby HBV affects NFAT5 through separating the upstream pathway and convergent downstream pathways of NFAT5 in hepatoma cells. We found that HBV suppressed NFAT5 expression by inducing hypermethylation of the AP1-binding site in the NFAT5 promoter. Though HBV doesn’t encode any methylases, however HBV infection can stimulate the overexpression of DNMTs, particularly DNMT1, DNMT3A and DNMT3B [25]. We speculate the overexpression of DNMTs may result in the hyper-methylation of the AP1-binding site in the NFAT5 promoter. In addition HBV also inhibited NFAT5 through miR-30e-5p targeting of the MAP4K4 signaling pathway. DARS2 promoted HCC tumorigenesis by accelerating cell cycle progression and attenuating cell apoptosis, as a downstream target gene of NFAT5. Our results suggest that the upregulation of DARS2 by HBV promotes hepatocarcinogenesis through the miR-30e-5p/MAP4K4/NFAT5 pathway.

MicroRNAs (miRNAs) comprise a group of small noncoding RNAs regulating gene expression at the posttranslational level, thereby participating in fundamental biological processes, including cell proliferation, differentiation, and apoptosis [26]. Mounting evidence indicates that dysregulation of miRNAs plays important roles in HBV infection and HBV-associated HCC [27–30]. Here, by screening a series of miRNAs based on reports in the literature, we identified miR-30e-5p as an effective reinforcer of NFAT5. We discovered that miR-30e-5p was downregulated in HBV-associated HCC, acting as a tumor suppressor, and that HBV could suppress miR-30e-5p expression. Several studies support our findings. For example, miR-30e is expressed at significantly lower levels in the sera of HCC patients than in healthy volunteers, suggesting serum miR-30e as a novel noninvasive biomarkers of hepatocellular carcinoma [31]. miR-30e inhibits the proliferation of hepatoma cells through directly targeting the 3′-UTR of P4HA1 mRNA [32]. However, NFAT5 is not a target gene of miR-30e-5p according to prediction using TargetScan. The existing literature indicates that the p38/MAPK pathway is associated with the NaCl-induced nuclear translocation of NFAT5 [18, 33, 34]. Moreover, the TargetScan database indicated that MAP4K4 is a target gene of miR-30e-5p, which also participates in the MAPK signaling pathway. We hypothesized that the MAPK signaling pathway might be involved into the correlation between NFAT5 and miR-30e-5p. Thus, we performed Western blot assays of components of the MAPK signaling pathway under miR-30e-5p overexpression. The results showed that miR-30e-5p inhibited MAP4K4 expression and thereby suppressed the downstream protein c-MYC by decreasing the level of phosphorylated ERK1/2 in Hep3B cells. We also found that miR-30e-5p inhibited MAP4K4 by binding to the 3128 bp–3135 bp positions of the MAP4K4 3′-UTR in luciferase reporter assays, which indicated that MAP4K4 is a direct target gene of miR-30e-5p. We then focused on c-MYC, since it can bind to the NFAT5 promoter according to ALGGEN PROMO, and c-MYC is downstream of the MAPK signaling pathway according to the KEGG database. We next studied the mechanism of interaction between c-MYC and NFAT5 via ChIP-PCR. The results showed that c-MYC inhibited NFAT5 transcription by binding to the NFAT5 promoter (positions −observed to be upregulated when we treated Hep3B cells with 10,058-F4, a c-MYC inhibitor, indicating that c-MYC inactivates NFAT5 transcription via binding to its promoter. These results suggest that HBV inhibits NFAT5 expression through miR-30e-5p targeting of the MAP4K4 signaling pathway.

To further study the molecular mechanism underlying the promotion of hepatoma cell apoptosis by NFAT5, we identified targeted proteins associated with NFAT5 function to illustrate the role of the downstream pathway of NFAT5 in HCC. The combination of ChIP and next-generation sequencing is a biochemical technique used to identify direct gene targets of a factor of interest in a genome-wide, unbiased manner and to elucidate their functional significance [35]. Novel targets of NFAT5 were revealed using ChIP-sequencing, as shown in Table 2. Among these targeted genes, DUSP5P and UBE2MP1 are lncRNAs and were therefore excluded from further analysis. A literature review of the other 5 targets showed that some of these genes have been previously studied in tumor diseases. RNF126 promotes the proliferation and viability of tongue cancer by regulating the AKT signaling pathway [36]. RNF126 was found to positively regulate BRCA1 by directly interacting with E2F1 for homologous recombination in breast and ovarian cancer [37]. WRNIP1 is involved in cell cycle progression, and its phosphorylation is reduced by FGFR1OP in lung cancer [38]. However, DARS2 has not been previously studied in any tumor disease. The DARS2 gene is located on chromosome 1q25.1 and encodes mitochondrial aspartyl-tRNA synthetase, which is important for the mitochondrial unfolded protein response (UPRmt) [39]. Mutations in the DARS2 gene are associated with leukoencephalopathy (LBSL) [19, 20]. To study the relationship between NFAT5 and DARS2 in HBV-associated HCC, we investigated the effect of HBV on NFAT5 and DARS expression using HepG2.2.15 cells carrying an integrated fragment of HBV genomic DNA and HepG2 cells that did not carry HBV genomic DNA. The results showed that HBV upregulated DARS2 expression and inhibited NFAT5 expression simultaneously. We next found that NFAT5 negatively regulated DARS2, and the inhibition of tumorigenesis by NFAT5 on was executed through DARS2 in different hepatoma cell lines. Subsequent experiments showed that DARS2 expression was higher in HCC tissues than in para-tumor tissues and that DARS2 regulated the cell cycle progression and apoptosis of HCC cells. The present study revealed that aberrant expression of DARS2 contributed to HCC development, and DARS2 may be a potential target for the treatment and diagnosis of HCC. Although DARS2 has not been identified in previous tumor studies, its biological function is well documented. DARS2 depletion leads to severe deregulation of mitochondrial protein synthesis, followed by a large mitochondrial respiratory chain (MRC) deficit [40], indicating that DARS2 upregulation could contribute to higher mitochondrial efficiency. The mitochondrial-dependent apoptosis pathway is initiated by cyclophilin-D, leading to reduction of the mitochondrial membrane potential (MMP) and, ultimately, opening of the mitochondrial permeability transition pore (mPTP) [41]. A recent study showed that ECHS1 binding to HBsAg decreased MMP, inducing mitochondrial-dependent apoptosis [42]. Our clinical data indicated that DARS2 was associated with HBV infection, and DARS2 might therefore be associated with ECHS1. Thus, we hypothesize that DARS2 positively regulates mitochondrial function and induces MMP, contributing to the growth of HCC cells. However, this hypothesis requires verification through further research. The available evidence suggests that DARS2 plays an essential role in mitochondrial function. However, the mechanism underlying the regulation of mitochondrial function by DARS2 remains unclear. Overall, how DARS2 promotes HCC tumorigenesis is a topic that deserves further study.

In summary, the present study illustrates that the upregulation of DARS2 by HBV promotes hepatocarcinogenesis through the miR-30e-5p/MAPK/NFAT5 pathway. Furthermore, NFAT5 acts as a tumor suppressor in HBV-associated HCC tissues by suppressing DARS2 expression. As aberrant expression of DARS2 contributes to HCC development, DARS2 may be a potential target for the treatment and diagnosis of HCC. Our results suggest that two pathway of inhibition on NFAT5 expression mediated by HBV may play an important role in the progression of HBV-associated HCC. These findings provide new insights that increase our understanding of the molecular mechanisms involved in the development of HBV-associated HCC via the miR-30e-5p/MAPK/NFAT5/DARS2 pathway.

## Additional files

Additional file 1: Tables S1-S3. (**Table S1**) Relationship between NFAT5 expression and clinicopathologic parameters of HCC patients. (**Table S2 **) Unknown target genes of NFAT5 detected by ChIP-Seq. (**Table S3**) Univariate and multivariate cox regression analysis of DARS2. (DOCX 18 kb)

Additional file 2: Figure S1. **(A)** NFAT5 was downregulated in HBV-associated HCC tissue compared with non-viral HCC tissue, confirmed by IHC. **(B)** Bioinformatics analyses of methylation sites of CpG island of NFAT5 promoter showed AP1 binding site was located in CpG island. **(C)** Left panel: Luciferase activity of NFAT5 promoter was tested in Huh7 cells transfected with the plasmid pBlue-HBV treated with 5-Aza-CdR (DNA methylation inhibitor) for 72 h. Right panel: NFAT5 expression was measured by RT-qPCR, followed by MSP analysis in the NFAT5 CpG sites in Huh7 cells treated with 5-Aza-CdR for 72 h. **(D)** MiR-30e-5p expression in 10 pairs of tissue were displayed. **(E)** HBsAg (left panel) and HBeAg (right panel) of HepG2.2.15 medium was detected by Roche cobas 4000 with technique of electrochemiluminescence immunoassay. **(F)** MiR-30e-5p inversely mediated HBx expression and reduced its expression in turn, both in Hep3B and HepG2.2.15. **(G)** MiR-30e-5p bound to the MAP4K4 3’UTR at position 3128–3135, as predicted by TargetScan. **(H)** Wild-type and mutated MAP4K4 3’UTR sequences were designed for luciferase reporter assays. **(I)** The KEGG database showed that MAP4K4 is involved in the MAPK signaling pathway, inducing c-MYC and the phosphorylation of ERK1/2. Arrows with 2 transverse lines represent inhibition, and arrows with +p represent inducing phosphorylation. **(J)** The c-MYC protein bound to position -1396 bp~ − 1364 bp of the NFAT5 promoter, as predicted by ALGGEN PROMO. **(K)** DARS2 expression in 15 pairs of tissues with typical difference is shown. *P < 0.05, **P < 0.01, ***P < 0.0001 (PDF 20127 kb)

Additional file 3: Figure S2. **(A)** Tumors with a diameter ≥ 2 cm had higher DARS2 expression than did tumors smaller than 2 cm (*P* = 0.0299). **(B)** DARS2 expression was upregulated in patients with HBV infection compared with that in patients not infected with HBV (P < 0.0001). **(C)** DARS2 expression in HCC patients at different BCLC stages. DARS2 expression in stage 0-A patients was not significantly different from that in patients in stage B (*P* = 0.5338) but was lower than that in patients in stage C (*P* = 0.004) and stage D (*P* < 0.0001). Other comparisons not shown are as follows: *P* = 0.0145 for stage B vs stage C, *P* < 0.0001 for stage B vs stage D, and *P* = 0.0494 for stage C vs stage D. **(D)** DARS2 was associated with HCC cell differentiation. Patients with poor differentiation had significantly higher DARS2 expression than did patients with well-differentiated (P < 0.0001) and moderately differentiated (*P* = 0.0358) tumors. Patients with moderate differentiation also expressed more DARS2 than did those with well-differentiated tumors (*P* = 0.0423). **(E)** HCC patients with distal metastasis expressed higher levels of DARS2 than did patients without metastasis (*P* = 0.0166). **(F)** Patients with portal vein invasion had higher DARS2 expression than did patients without portal vein invasion (*P* = 0.0011) (PDF 3716 kb)

## Abbreviation

3’UTR: 3′ untranslated region
AFP: Alpha-feroprotein
AGE: Agarose gel electrophoresis
AUC: Area under curve
cDNA: Circle DNA
ChIP-Seq: Chromatin immunoprecipitation and the next generation of sequencing
EGFR: Epidermal growth factor receptor
EMT: Epithilial-mesenchymal transition
FBS: Fetal bovin serum
FCM: Flow cytometry
HBeAg: Hepatitis B e antigen
HBsAg: Hepatitis B surface antigen
HBV: Hepatitis B virus
HCC: Hepatocellular carcinoma
HR: Hazard ratio
IHC: Immunohistochemical staining
KM: Kaplan-Meier survival analysis
LINEs: Long interspersed nuclear elements
lncRNA: Long non-coding RNA
MD: Mean of difference
miRNAs: Micro-RNAs
MRC: Mitochrondrial respiratory chain
NC: Negative control
NFAT5: Nuclear factor of activated T cells 5
NT: Non-tumor/Para-tumor
OREBP: Osmotic response element binding protein
PARP: Poly-ADP-ribose polymerase
PMSF: Phenylmethanesulfonyl fluoride
ROC curve: Receiver operating characteristic curve
RT-qPCR: Real-time quantitative polymerase chain reaction
SAMe: S-adenosylmethionine
SDS-PAGE: Sodium dodecyl sulfate polyacrylamide gel electrophoresis
SEM: Standard error of mean
siRNA: Small interfering RNA
T: Tumor
TG: Transfection group
TonEBP: Tonicity-responsive enhancer binding protein
VEGF: Vascular endothelial growth factor
WB: Western blot assay
