# Supplementary material for: Upregulation of DARS2 by HBV promotes hepatocarcinogenesis through the miR-30e-5p/MAPK/NFAT5 pathway
Source: J Exp Clin Cancer Res. 2017 Oct 19;36:148. doi: 10.1186/s13046-017-0618-x (PMC5649064; doi:10.1186/s13046-017-0618-x)
Supplement: Supplementary file 1 — (Table S1) Relationship between NFAT5 expression and clinicopathologic parameters of HCC patients. (Table S2 ) Unknown target genes of NFAT5 detected by ChIP-Seq. (Table S3) Univariate and multivariate cox regression analysis of DARS2. (DOCX 18 kb) [file 13046_2017_618_MOESM1_ESM.docx]

**Table S1 Relationship between NFAT5 expression and clinicopathologic parameters of HCC patients**

| Characteristics | Number of cases | NFAT5 expression | | | | P value |
| --- | --- | --- | --- | --- | --- | --- |
|  |  | Low（n=45） | | High（n=45） | |  |
| Gender |  |  |  |  |  | 0.352 |
| Male | 78 | 37 | | 41 | |  |
| Female | 12 | 8 | | 4 | |  |
| Age(years) |  |  |  |  |  | 0.393 |
| >55 | 38 | 21 | | 17 | |  |
| ≤55 | 52 | 24 | | 28 | |  |
| Tumor size |  |  |  |  |  | 0.829 |
| ≥5cm | 55 | 27 | | 28 | |  |
| <5cm | 35 | 18 | | 17 | |  |
| Histologic grade |  |  |  |  |  | 0.035 |
| Well or moderate | 74 | 33 | | 41 | |  |
| Poor | 16 | 12 | | 4 | |  |
| BCLC stage |  |  |  |  |  | <0.01 |
| A | 57 | 15 | | 42 | |  |
| B+C | 33 | 30 | | 3 | |  |
| HBV infection |  |  |  |  |  | <0.01 |
| Postive | 69 | 43 | | 26 | |  |
| Negtive | 21 | 2 | | 19 | |  |
| Liver cirrhosis |  |  |  |  |  | <0.01 |
| Yes | 56 | 42 | | 14 | |  |
| No | 34 | 3 | | 31 | |  |
| Serum AFP(μg/L) |  |  |  |  |  | 0.090 |
| ≥400 | 67 | 37 | | 30 | |  |
| <400 | 23 | 8 | | 15 | |  |

**Table S2 Unknown target genes of NFAT5 detected by ChIP-Seq**

| Gene ID | TSID | Peak location | Peaks annotations | Gene Descriptions |
| --- | --- | --- | --- | --- |
| CENPL | NM_033319,NM_001127181,NM_001171182 | chr1-172060625-172060701 | Promoter | Centromere Protein L |
| RNF126 | NM_194460 | chr19:596967-597166 | Downstream extremity | RING Finger Protein 126 |
| ZNF555 | NM_001172775,NM_152791 | chr19:2793373-2793431 | Promoter, Introns, | Zinc Finger Protein 555 |
| DARS2 | NM_018122 | chr1:172060625-172060701 | Promoter | Aspartyl-tRNA Synthetase, Mitochondrial |
| WRNIP1 | NM_130395,NM_020135 | chr6:2709046-2709249 | Promoter | ATPase Werner Helicase Interacting Protein 1 |
| DUSP5P | NR_002834 | chr1:226831554-226831603 | Distal | Dual Specificity Phosphatase 5 Pseudogene |
| UBE2MP1 | NR_002837 | chr16:34152879-34153102 | Introgenic | Ubiquitin Conjugating Enzyme E2 M Pseudogene 1 |

**Table S3 Univariate and multivariate cox regression analysis of DARS2**

| Variable | Univariate analysis | |  | Multivariate analysis | |
| --- | --- | --- | --- | --- | --- |
|  | HR (95%CI) | P value |  | HR (95%CI) | P value |
| DARS2 | 3.559(1.114-11.372) | **0.032** |  | 4.448(1.581-12.519) | **0.005** |
| Tumor size | 1.152(0.596-2.227) | 0.674 |  | - | - |
| HBV | 1.391(0.608-3.185) | 0.435 |  | - | - |
| BCLC Stages | 1.859(1.256-2.753) | **0.002** |  | 1.579(1.121-2.225) | **0.009** |
| Differentiation | 1.739(1.132-2.670) | **0.012** |  | 1.678(1.136-2.480) | **0.009** |
| Liver cirrhosis | 0.522(0.244-1.118) | 0.094 |  | - | - |
| Intrahepatic metastasis | 0.936(0.436-2.009) | 0.865 |  | - | - |
| Distal metastasis | 0.386(0.140-1.063) | 0.065 |  | - | - |
| Portal vein invasion | 2.382(1.096-5.178) | **0.029** |  | 1.912(0.906-4.036) | 0.089 |
| Serum AFP | 1.031(0.612-1.738) | 0.908 |  | - | - |
