# Supplementary material for: Upregulation of DARS2 by HBV promotes hepatocarcinogenesis through the miR-30e-5p/MAPK/NFAT5 pathway
Source: J Exp Clin Cancer Res. 2017 Oct 19;36:148. doi: 10.1186/s13046-017-0618-x (PMC5649064; doi:10.1186/s13046-017-0618-x)

Additional file 1: Figure S1

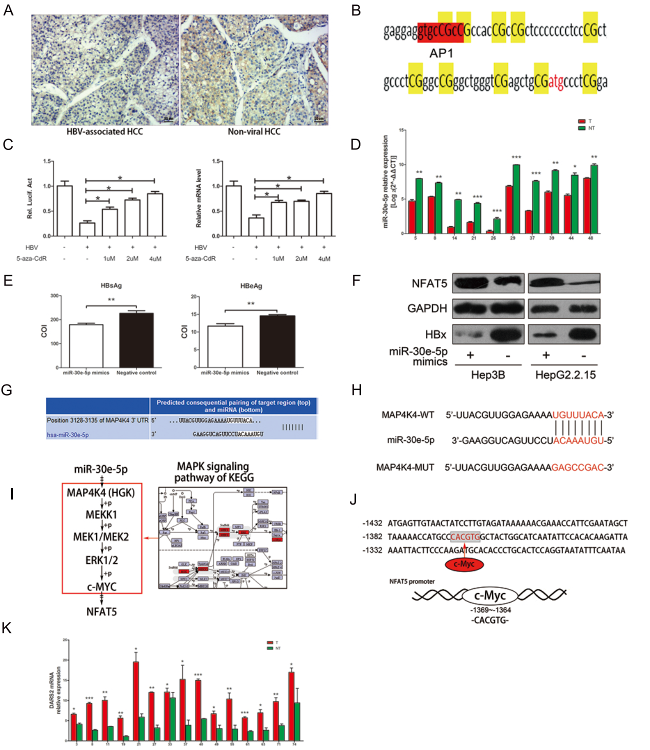

Supplement: Supplementary file 2 — (A) NFAT5 was downregulated in HBV-associated HCC tissue compared with non-viral HCC tissue, confirmed by IHC. (B) Bioinformatics analyses of methylation sites of CpG island of NFAT5 promoter showed AP1 binding site was located in CpG island. (C) Left panel: Luciferase activity of NFAT5 promoter was tested in Huh7 cells transfected with the plasmid pBlue-HBV treated with 5-Aza-CdR (DNA methylation inhibitor) for 72 h. Right panel: NFAT5 expression was measured by RT-qPCR, followed by MSP analysis in the NFAT5 CpG sites in Huh7 cells treated with 5-Aza-CdR for 72 h. (D) MiR-30e-5p expression in 10 pairs of tissue were displayed. (E) HBsAg (left panel) and HBeAg (right panel) of HepG2.2.15 medium was detected by Roche cobas 4000 with technique of electrochemiluminescence immunoassay. (F) MiR-30e-5p inversely mediated HBx expression and reduced its expression in turn, both in Hep3B and HepG2.2.15. (G) MiR-30e-5p bound to the MAP4K4 3’UTR at position 3128–3135, as predicted by TargetScan. (H) Wild-type and mutated MAP4K4 3’UTR sequences were designed for luciferase reporter assays. (I) The KEGG database showed that MAP4K4 is involved in the MAPK signaling pathway, inducing c-MYC and the phosphorylation of ERK1/2. Arrows with 2 transverse lines represent inhibition, and arrows with +p represent inducing phosphorylation. (J) The c-MYC protein bound to position -1396 bp~ − 1364 bp of the NFAT5 promoter, as predicted by ALGGEN PROMO. (K) DARS2 expression in 15 pairs of tissues with typical difference is shown. *P < 0.05, **P < 0.01, ***P < 0.0001 (PDF 20127 kb) [file 13046_2017_618_MOESM2_ESM.pdf]
