# Supplementary figures and images for: Upregulation of DARS2 by HBV promotes hepatocarcinogenesis through the miR-30e-5p/MAPK/NFAT5 pathway
Source: J Exp Clin Cancer Res. 2017 Oct 19;36:148. doi: 10.1186/s13046-017-0618-x (PMC5649064; doi:10.1186/s13046-017-0618-x)

# Additional file 2: Figure S2

A

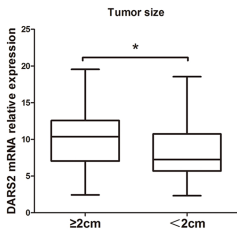

B

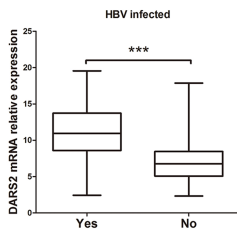

C

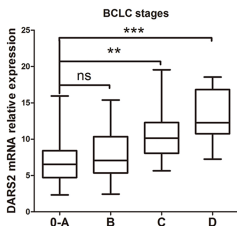

D

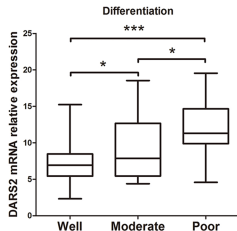

E

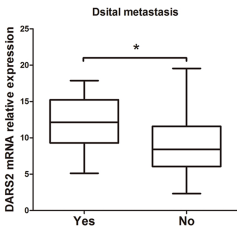

F

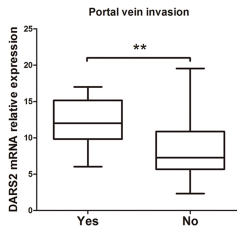

Supplement: Supplementary file 3 — (A) Tumors with a diameter ≥ 2 cm had higher DARS2 expression than did tumors smaller than 2 cm (P = 0.0299). (B) DARS2 expression was upregulated in patients with HBV infection compared with that in patients not infected with HBV (P < 0.0001). (C) DARS2 expression in HCC patients at different BCLC stages. DARS2 expression in stage 0-A patients was not significantly different from that in patients in stage B (P = 0.5338) but was lower than that in patients in stage C (P = 0.004) and stage D (P < 0.0001). Other comparisons not shown are as follows: P = 0.0145 for stage B vs stage C, P < 0.0001 for stage B vs stage D, and P = 0.0494 for stage C vs stage D. (D) DARS2 was associated with HCC cell differentiation. Patients with poor differentiation had significantly higher DARS2 expression than did patients with well-differentiated (P < 0.0001) and moderately differentiated (P = 0.0358) tumors. Patients with moderate differentiation also expressed more DARS2 than did those with well-differentiated tumors (P = 0.0423). (E) HCC patients with distal metastasis expressed higher levels of DARS2 than did patients without metastasis (P = 0.0166). (F) Patients with portal vein invasion had higher DARS2 expression than did patients without portal vein invasion (P = 0.0011) (PDF 3716 kb) [file 13046_2017_618_MOESM3_ESM.pdf]
